# Scavenger receptor CD36 governs recruitment of myeloid cells to the blood–CSF barrier after stroke in neonatal mice

**DOI:** 10.1186/s12974-022-02388-z

**Published:** 2022-02-11

**Authors:** Aditya Rayasam, Amin Mottahedin, Joel Faustino, Carina Mallard, Zinaida S. Vexler

**Affiliations:** 1 Department of Neurology, University California San Francisco, 675 Nelson Rising Lane, San Francisco, CA 94158-0663 USA; 2 Department of Physiology, Institute of Neuroscience and Physiology, Sahlgrenska Academy, University of Gothenburg, Gothenburg, Sweden

**Keywords:** Middle cerebral artery occlusion, Neonate, Inflammation, Extracellular matrix, Choroid plexus

## Abstract

**Background:**

Ischemic stroke induces the activation and recruitment of peripheral leukocytes to the injured brain. These cells can infiltrate the brain through multiple routes, either by penetrating blood–brain barrier or via blood–CSF barriers at the meninges or the choroid plexus (CP). We previously showed that myeloid cell trafficking via the CP occurs early after neonatal arterial stroke and modulates injury. CD36 is a receptor that mediates function of endothelial cells and cells of the monocyte lineage under various neurodegenerative conditions and can influence brain injury after neonatal stroke. Here we asked whether CD36 impacts injury by altering leukocyte trafficking through the CP in neonatal mice subjected to transient middle cerebral artery occlusion (tMCAO).

**Methods:**

In neonatal mice with intact or globally disrupted CD36 signalling (CD36 KO), we characterized the phenotypes of myeloid cells by flow cytometry and the underlying gene expression signatures in the CPs contralateral and ipsilateral to tMCAO by RNA sequencing analyses, focussing on early post-reperfusion time window.

**Results:**

Flow cytometry in the isolated CPs revealed that CD36 mediates stepwise recruitment of myeloid cells to the CP ipsilateral to tMCAO early after reperfusion, with a predominant increase first in inflammatory monocyte subsets and neutrophils followed by patrolling monocytes. RNA sequencing analyses demonstrated marked changes in gene expression in the CP ipsilateral compared to the CP contralateral to tMCAO in wild type mice. Changes were further modified by lack of CD36, including distinction in several clusters of genes involved in inflammatory, metabolic and extracellular matrix signalling in the CP ipsilateral to tMCAO.

**Conclusion:**

Altogether, our data suggest cooperation between blood–CSF–brain interface via the CP through CD36-mediated signalling following neonatal stroke with a key role for inflammatory monocytes and neutrophils.

**Supplementary Information:**

The online version contains supplementary material available at 10.1186/s12974-022-02388-z.

## Introduction

Post-stroke inflammation is a double-edged sword in brain injury and involves various cellular players within the brain and also infiltrating immune cells [1]. Microglia cells are the main professional immune cells resident in the brain and promptly react to cerebral ischemia, as identified by changes in morphology [2] and inflammatory cytokine release [3]. However, depletion of microglia exacerbates stroke outcome in adult [4, 5] and neonatal mice [6], suggesting a beneficial role for these cells, in part by removal of dead cells by phagocytosis and production of neurotrophic factors [1]. Infiltrating immune cells, in turn, are diverse after stroke, with discrepant spatial–temporal effects [7]. Despite decades of studies, the route and mechanisms of leukocyte infiltration into the brain remain to be fully understood. Moreover, we and others have shown major differences in inflammatory response [8] and blood–brain barrier (BBB) function [9] early after neonatal and adult stroke. However, data on cellular and molecular mechanisms of neonatal stroke remain scarce.

CD36, a type B scavenger receptor, is a multifunctional transmembrane protein expressed in many cell types including, microglia, monocytes, macrophages, epithelial and endothelial cells [10]. CD36 exerts multiple biological functions by mediating innate immunity, fatty acid transport and lipid signalling, assembly of inflammatory pathways in lipid rafts and ROS production [11]. CD36 also mediates several steps in phagocytosis of apoptotic material, including recognition of apoptotic cells, engulfment and digestion of apoptotic bodies [12–14]. Furthermore, CD36 serves as an anti-angiogenic factor and is involved in enhancement of platelet aggregation [15]. CD36-mediated effects are ligand-specific and context-dependent [10, 16] and are mediated via its cooperation with other receptors, including vitronectin and Toll-like receptors (TLR), TLR2, TLR4 and TLR6 [17, 18]. CD36 utilizes multiple ligands, such as phospholipids, advanced glycation end products, TSP-1 and oxidized low-density lipoprotein (OxLDL). In stroke and under neurodegenerative conditions in the adult and ageing brain, CD36 has been found harmful (reviewed in [19]). While the presence of CD36 exacerbates injury after acute transient middle cerebral artery occlusion (tMCAO) in the adult [13], in neonatal mice we observed much higher incidence of severe injury after acute tMCAO in mice that lack CD36 [20]. Many CD36 ligands are low at birth, likely contributing to the differing CD36 effects between neonatal and adult brain. Thus far we identified several divergent aspects of CD36 signalling in neonates compared to adults after tMCAO. While superoxide accumulation was largely responsible for injury in the adult [13], we observed similar superoxide accumulation in the vessels and in macrophages in ischemic-reperfused regions of acutely injured WT and CD36 KO neonates [21]. At the same time, we showed that lack of CD36 in injured neonatal mice affects microglial morphology, limits phagocytosis and reshapes intracellular lipid signalling dependent on a Src kinase Lyn [21].

Knowing that CD36 plays a key role in shaping macrophage phenotypes after injury [14, 22, 23] and that the choroid plexus (CP) serves as a gateway for early leukocyte accumulation after neonatal stroke [24], we examined effects of global CD36 knockout on leukocyte trafficking and the transcriptome in the CPs after neonatal stroke. We demonstrate that CD36 mediates recruitment of multiple leukocyte subtypes to the CP ipsilateral to tMCAO early after reperfusion, potentially contributing to increased number of CD45^high^CD11b^+^ cells in ischemic-reperfused brain parenchyma. tMCAO triggers marked changes in gene expression in the CP of WT pups, including activation of genes involved in inflammatory, metabolic and extracellular matrix signalling, changes that are further modified by lack of CD36.

## Materials and methods

### Animals

All research conducted on animals was approved by the University of California San Francisco Institutional Animal Care and Use Committee and in accordance with the Guide for the Care and Use of Laboratory Animals (U.S. Department of Health and Human Services). Animals were given ad libitum access to food and water, housed with nesting material and shelters, and kept in rooms with temperature control and light/dark cycles. The data are in compliance with the ARRIVE guidelines (Animal Research: Reporting in Vivo Experiments). Block litter design and randomization within individual litters were used. Blinded data analysis was used where possible.

### Transient middle cerebral artery occlusion (tMCAO)

tMCAO was performed on postnatal day 9 (P9)-P10 C57BL/6 wild type (WT; purchased from Charles River) mice and CD36 KO mice of both sexes, as we previously described [21]. Briefly, a midline cervical incision was made under isoflurane anesthesia, the common carotid artery and internal carotid artery (ICA) exposed, single threads from a 7-0 silk suture used to temporary tie a knot below the origin of the ICA to prevent retrograde bleeding from the arteriotomy. A coated 8-0 nylon suture was advanced 4–5 mm and removed 3 h later. In sham-operated pups suture was inserted but not advanced. Mice from the same litters were randomized to receive tMCAO or sham surgery. Temperature was maintained with temperature controlled blanket and overhead lamp. Based on our historic diffusion-weighted MRI data in the model and the presence of recirculation upon suture removal, as evident using intra-jugular injection of FITC–isolectin B4 [6, 21, 25], the incidence of injury is > 70% and no bleeding associated with reperfusion. Data for male and female pups were combined based on our published data on similar injury in male and female pups at 72 h [25] and unpublished data for outcomes at 1–4 weeks of reperfusion in several mouse lines on C57BL/6 background.

### Histology and immunofluorescence

Animals were perfused and post-fixed with 4% PFA. Post-fixed, cryoprotected and flash frozen brains were sectioned on a cryostat (12 μm thick serial sections, 360 μm apart). Double-immunofluorescence was performed on adjacent sections blocked in 10% NGS/PBST and incubated overnight in 2% NGS/PBST with rabbit anti-mouse GLUT-1 (1:500, AbCAM), rabbit anti-Iba1 (1:500, WAKO), anti-mouse Timp1 (1:200, TFS) followed by appropriate secondary antibodies purchased from Invitrogen and DAPI. Z-stacks of 10–12 images were captured at 1.0 μm intervals (25 ×/100 × oil objectives, Zeiss Axiovert 100 equipped with Volocity Software, Improvision/Perkin Elmer) and analysis performed in four–five fields of view (FOV) per hemisphere/region in the ischemic-reperfused cortex and matching contralateral tissue using automated protocols for signal intensity threshold (> 2SD background in each channel) in a 1 × 10^6^μm^3^ voxel.

### Myeloid cell isolation

Mice deeply anesthetized with isoflurane were transcardially perfused with cold PBS, red blood cells were lysed using ACK lysis buffer, and cells were washed with RPMI. Brain and CP tissue were minced with razor blades and pushed through 70 μm nylon cell strainers, placed in 1 mg/ml collagenase for 45 min at 37 °C inverted frequently. Cells from brain tissue were washed, resuspended in 70% Percoll and overlaid with 30% Percoll. The cells were centrifuged at 2400 rpm for 30 min at 4 °C without brake. The interface was removed and washed before plating.

### Flow cytometry

Single-cell myelin-free suspensions from contralateral and injured regions were plated (5 × 10^5^/96 well), centrifuged, pellet resuspended in 100 μl blocking buffer containing CD16/32 (1:70, Biolegend) and incubated in 150 μl FACs staining buffer containing 2% FBS. For intracellular cytokine staining, cells were fixed (Fixation and Permeabilization kit, BD Bioscience), incubated with antibody mixture on ice for 20 min, washed, centrifuged, resuspended in staining buffer and evaluated on BD LSRII flow cytometer (BD Biosciences). Fluorescence Minus One (FMO) samples, a commonly used strategy to prevent false positive results through overlap of fluorophores [26], was applied. The following combinations of antibodies diluted 1:200 in FACS staining buffer were used: anti-CD45-Pacific Blue (Biolegend), anti-CD11b-APC-Cy7 (Biolegend), Ly6g (IA8)-AF700 (Biolegend), Ly6c (Hk1.4)-APC (Biolegend), CD206-FITC (Biolegend), IL-10-PE-Cy7 (Biolegend), CD86-FITC (Biolegend), IL-1β-PE (Biolegend). Compensation beads (BD CompBeads) were incubated in Fixation and Permeabilization solution (100 μl, 4 °C, 20 min), incubated with antibody mixture (4 °C, 30 min) and resuspended in staining buffer. Gating and data analysis were performed using FlowJo software (Tree Star).

### Western blot

Western blot analysis was performed in cortical lysates using anti-Timp1 (1:200), TLR4 (1:200) and β-actin (1:5000 Sigma Aldrich) antibodies diluted in 5% milk in 0.2% Tween 20/TBS, 4 °C, overnight.

### RNA sequencing of the CP

The RNA extraction and RNA sequencing process was performed as described previously [27]. Briefly, the CP of mice subjected to tMCAO were collected from ipsilateral and contralateral side of the brain 3 h after reperfusion. After collection, CPs were placed in RNAlater (Ambion) for 24 h at 4 °C to preserve the RNA. Following a brief centrifuge, the RNAlater was removed and the CPs were stored at − 80 °C. Total RNA was extracted using Trizol (Qiazol,Qiagen) and miRNAeasy Kit (Qiagen) following manufacturer protocols. RNA quality was assessed using Experion Automated Electrophoresis System (Bio-Rad) and 500 ng of RNA was used for library preparation using TruSeq Stranded mRNA and Total RNA Sample Preparation Kit with Rib-Zero Gold (Illumina). The CPs from 5 mice were pooled together before RNA extraction. In sham-operated mice, ipsilateral and contralateral CP were mixed and samples from 3 mice were pooled. The sequencing was performed on a NovaSeq 6000 (Illumina) with a read length of 2 × 100. Mouse assembly mm10 from UCSC was used as reference genome.

### RNA sequencing data analysis

Differential expression was obtained using DEseq2 with Benjamini–Hochberg correction for multiple comparisons. Venny diagram v.2.1 was used for visualizing the number of differentially regulated genes in different groups. Gene cluster analysis was performed using the Qlucore Omics Analyser v.3.6 on top 1000 genes differentially regulated between groups (*q* < 0.05, *F* test and ANOVA). Enrichment analysis of gene clusters was performed using g:Profiler [28]. Ingenuity Pathway Analysis (IPA,Qiagen) was used to identify canonical pathways related to the regulated genes.

### Statistical analysis

Block litter design was used to avoid litter-to-litter variability, randomization for tMCAO/sham was used where possible, data for all figures were obtained on unsexed neonatal mice. Each dot on all graphs represents an individual mouse. Two-way *ANOVA* with post-hoc Turkey’s Multiple Comparison test was performed for comparing groups with multiple variables, as described in legends to individual figures. GraphPad Prism 8 software was utilized to generate statistical data. Differences were considered significant at *p* < 0.05. Results are shown as mean ± SD.

## Results

### Lack of CD36 alters myeloid cell recruitment to the ipsilateral cortex after tMCAO in neonatal mice

**Fig. 1:**
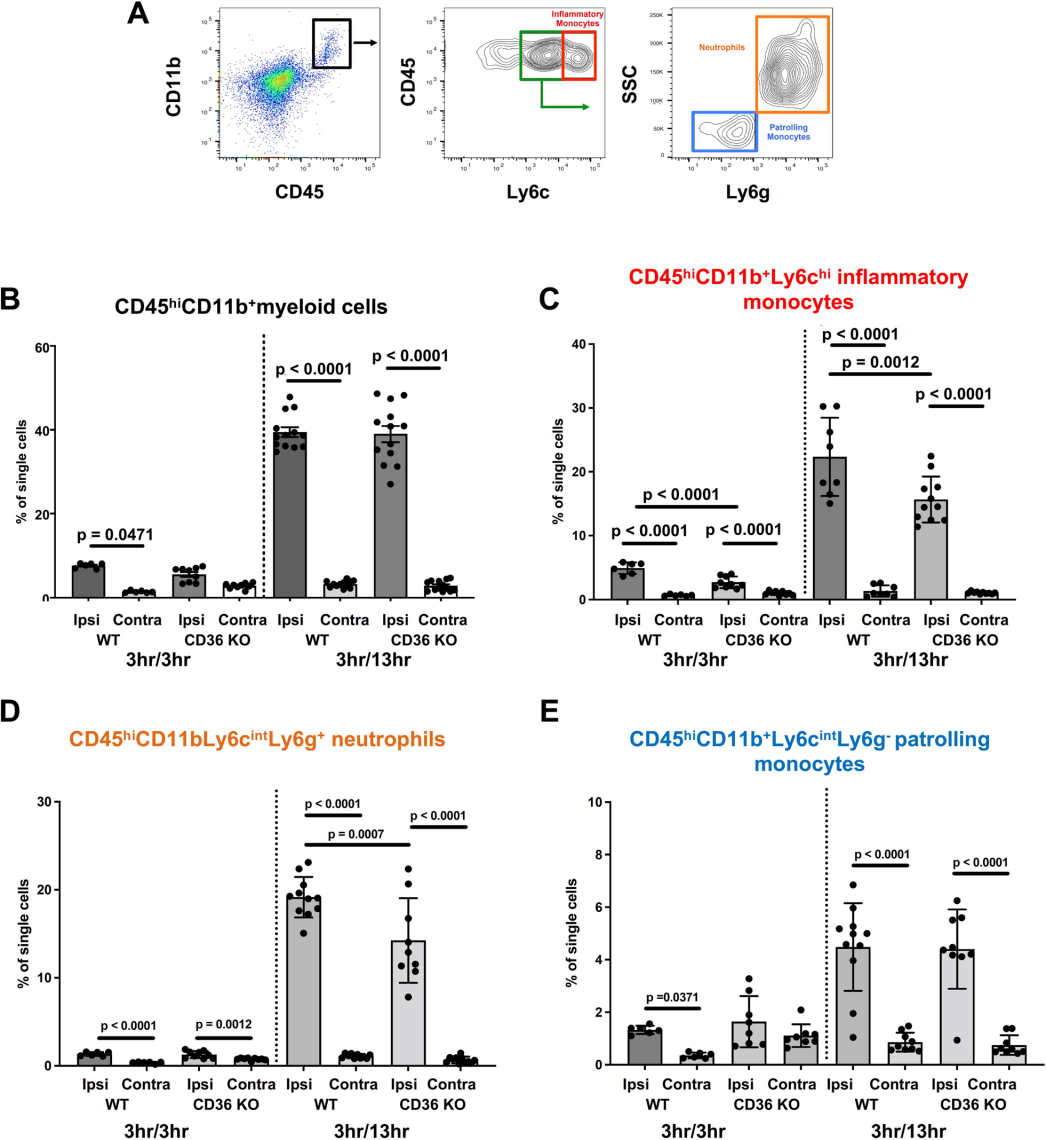
Lack of CD36 diminishes accumulation of myeloid cells in the ipsilateral cortex of neonatal mice subjected to tMCAO. **A** Gating strategy to identify subsets of myeloid cells from the dissociated cortices by flow cytometry. **B**–**E** Quantification of CD45^hi^CD11b^+^ myeloid cells (**B)**, CD45^hi^CD11b^+^Ly6c^hi^ inflammatory monocytes (**C)**, CD45^hi^11b^+^Ly6c^int^Ly6g^+^ neutrophils **(D)**, and CD45^hi^11b^+^Ly6c^int^Ly6g^−^ patrolling monocytes **(E)** ipsilateral (Ipsi) and contralateral (Contra) to tMCAO in WT and CD36 KO mice at 3 h and 13 h reperfusion. Two-way ANOVA with post-hoc Tukey’s Multiple Comparison test was performed to compare groups with multiple independent variables. Dots represent individual mice. Data are shown as mean ± SD. In this and the following figures 3 h/3 h refers to 3 h MCAO followed by 3 h reperfusion. 3 h/13 h refers to 3 h MCAO followed by 13 h reperfusion. Individual p values are listed within figures for data that are significant

First, based on our previous observations that genetic deletion of CD36 leads to much higher incidence of severe injury after acute neonatal stroke, in part due to disrupted immune signaling [21], we sought to determine whether microglial activation and accumulation of myeloid cells in the cortex after tMCAO in neonatal mice is regulated by CD36 signaling. We examined the presence of CD45^hi^CD11b^+^ cells in brain parenchyma and in matching contralateral regions at 3 h and 13 h (Fig. 1A), early post-reperfusion timepoints when transmigrated peripheral myeloid cells is the only population with high CD45 expression, while CD45 expression gradually rises over time in microglial cells [25].

Quantitative analysis of CD45^hi^CD11b^+^ cells revealed significantly increased accumulation of CD45^hi^CD11b^+^ cells in the ischemic-reperfused cortex relative to the contralateral cortex in WT pups but not in CD36 KO pups at 3 h (Fig. 1B). Significant increase of the number of CD45^hi^CD11b^+^ cells occurred in ischemic-reperfused regions of both WT and CD36 KO pups at 13 h, with no differences between the two groups (Fig. 1B). Analysis of the phenotypes of myeloid cells showed significantly more CD45^hi^CD11b^+^Ly6c^hi^ inflammatory monocytes in WT compared to CD36 KO mice at 3 h and at 13 h (Fig. 1C). Analysis of CD45^hi^11b^+^Ly6c^int^Ly6g^+^ neutrophils showed unchanged numbers early between genotypes but significant increase at 13 h reperfusion in WT ipsilateral compared to contralateral and ischemic-reperfused regions of CD36 KO mice (Fig. 1D). Both WT and CD36 KO mice exhibited significantly more CD45^hi^11b^+^Ly6c^int^Ly6g^−^ patrolling monocytes in the ischemic-reperfused cortex compared to the contralateral cortex at 13 h after reperfusion (Fig. 1E). Altogether, these results reveal that lack of CD36 modifies the pattern of myeloid cell infiltration by particularly muting the accumulation of inflammatory monocytes and neutrophils in the ischemic-reperfused cortex following tMCAO in neonatal mice,

### Lack of CD36 diminishes accumulation of myeloid cells in the ipsilateral CP of neonatal mice subjected to tMCAO

**Fig. 2:**
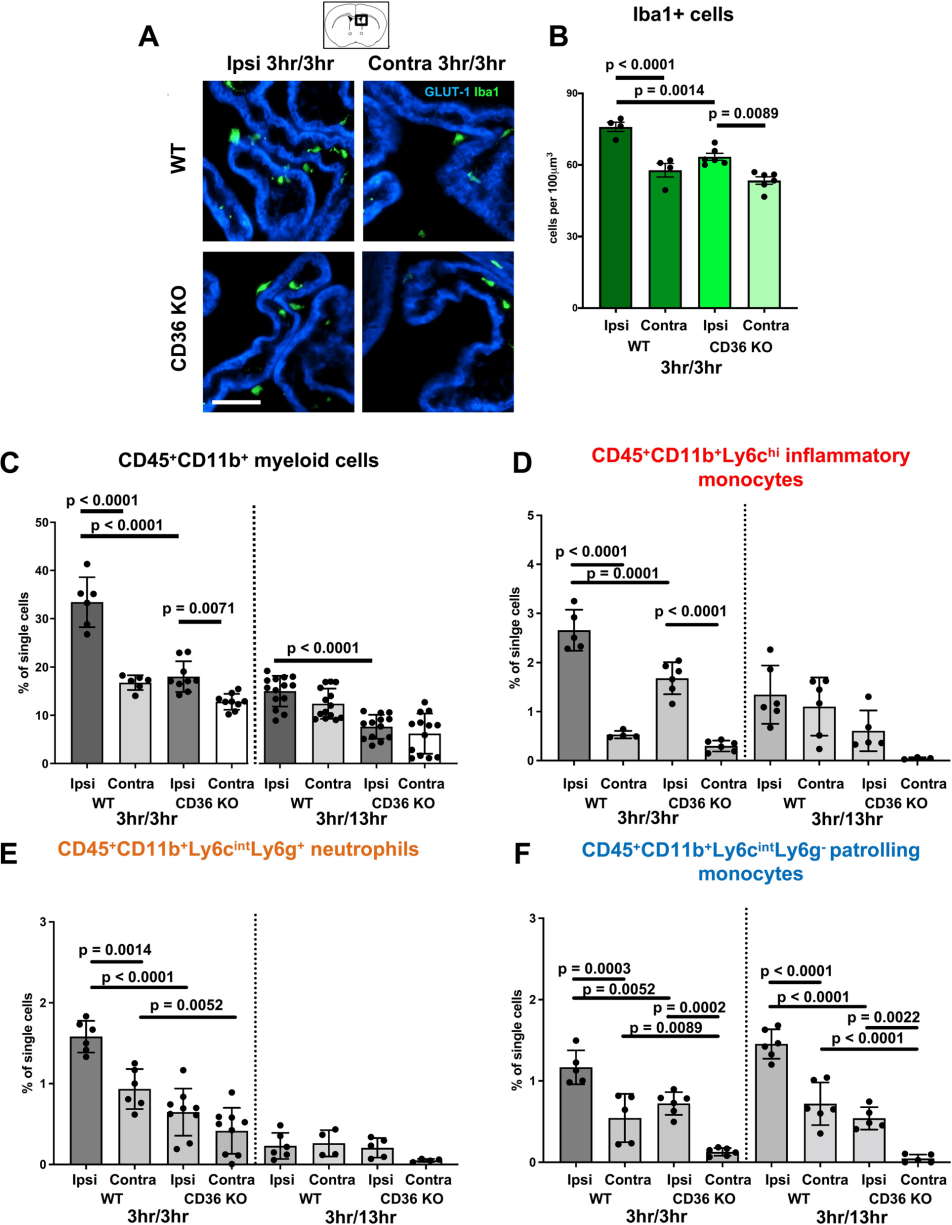
Accumulation of myeloid cells is attenuated in the ipsilateral CP of neonatal CD36 KO mice subjected to tMCAO. **A** Representative images of CPs at the level of the lateral ventricles immunostained with GLUT-1 (blue) and Iba1 (green) ipsilateral and contralateral to tMCAO in WT and CD36 KO mice at 3 h reperfusion. Scale bar = 50 µm. **B** Quantification of the number of Iba1^+^ cells in the ipsilateral and contralateral CPs in WT and CD36 KO mice at 3 h reperfusion**. C–F** Quantification of CD45^+^CD11b^+^ myeloid cells (**C**), CD45^+^CD11b^+^Ly6c^hi^ inflammatory monocytes (**D**), CD45^+^11b^+^Ly6c^int^Ly6g^+^ neutrophils (**E**), and CD45^+^11b^+^Ly6c^int^Ly6g^−^ patrolling monocytes (**F**) ipsilateral and contralateral to tMCAO in WT and CD36 KO mice at 3 h and 13 h reperfusion. Two-way ANOVA with post-hoc Tukey’s Multiple Comparison test was performed to compare groups with multiple independent variables (**B**–**F**). Dots represent individual mice. Data are shown as mean ± SD. Individual p values are listed within figures for data that are significant

Having previously established that the BBB is rather impermeable and neutrophil accumulation is markedly lower in neonates than in adults hours to days after tMCAO [6] and knowing leukocyte accumulation can occur rapidly in the ipsilateral CP following tMCAO in neonates [24], we examined the presence of cells of the monocyte lineage in the CPs at the level of the lateral ventricles by both immunofluorescence and flow cytometry. Quantification of Iba1^+^ cells in the ipsilateral and contralateral CPs (demarcated by GLUT-1^+^ to outline the CP vasculature, Fig. 2A) revealed significantly higher number of Iba1^+^ cells in the CP ipsilateral to tMCAO compared to the respective contralateral CP in WT and CD36 KO mice (Fig. 2A, B). However, the number of Iba1^+^ cells was significantly lower in the ipsilateral CP of CD36 KO mice compared to that in WT mice (Fig. 2A, B).

Flow cytometry in isolated CPs showed that in both WT and CD36 KO mice, the percentage of CD45^+^CD11b^+^ myeloid cells in the ipsilateral CP was significantly increased compared to respective contralateral CPs at 3 h after reperfusion (Fig. 2C), but significantly fewer CD45^+^CD11b^+^ myeloid cells were apparent in the ipsilateral CP of CD36 KO compared to WT mice. While the overall number of CD45^+^CD11b^+^ cells was reduced by 13 h reperfusion in the ipsilateral CP of both WT and CD36 KO mice, WT mice continued to display significantly more cells compared to CD36 KO mice in the ipsilateral CP. tMCAO increased CD45^+^CD11b^+^Ly6c^hi^ inflammatory monocytes and CD45^+^11b^+^Ly6c^int^Ly6g^+^ neutrophils in the ipsilateral CPs of both WT and CD36 KO mice (Fig. 2D, E) relative to contralateral CPs early on, yet by 13 h reperfusion, in both WT and CD36 KO mice, the influence of genotype and side relative to injury on inflammatory monocyte or neutrophil accumulation were not evident (Fig. 2D, E). Interestingly, the lack of CD36 influenced the numbers of CD45^+^11b^+^Ly6c^int^Ly6g^−^ patrolling monocytes at both 3 h and 13 h reperfusion, indicating delayed dynamic accumulation of these beneficial cells, which was particularly evident in WT mice (Fig. 2F). Cumulatively, these data demonstrate that genetic deletion of CD36 restricts myeloid cell accumulation in the CP ipsilateral to tMCAO and restricts the presence of both toxic and beneficial myeloid cells early after injury.

### tMCAO in neonatal WT mice induces marked early changes in gene expression in the CP ipsilateral to tMCAO

**Fig. 3:**
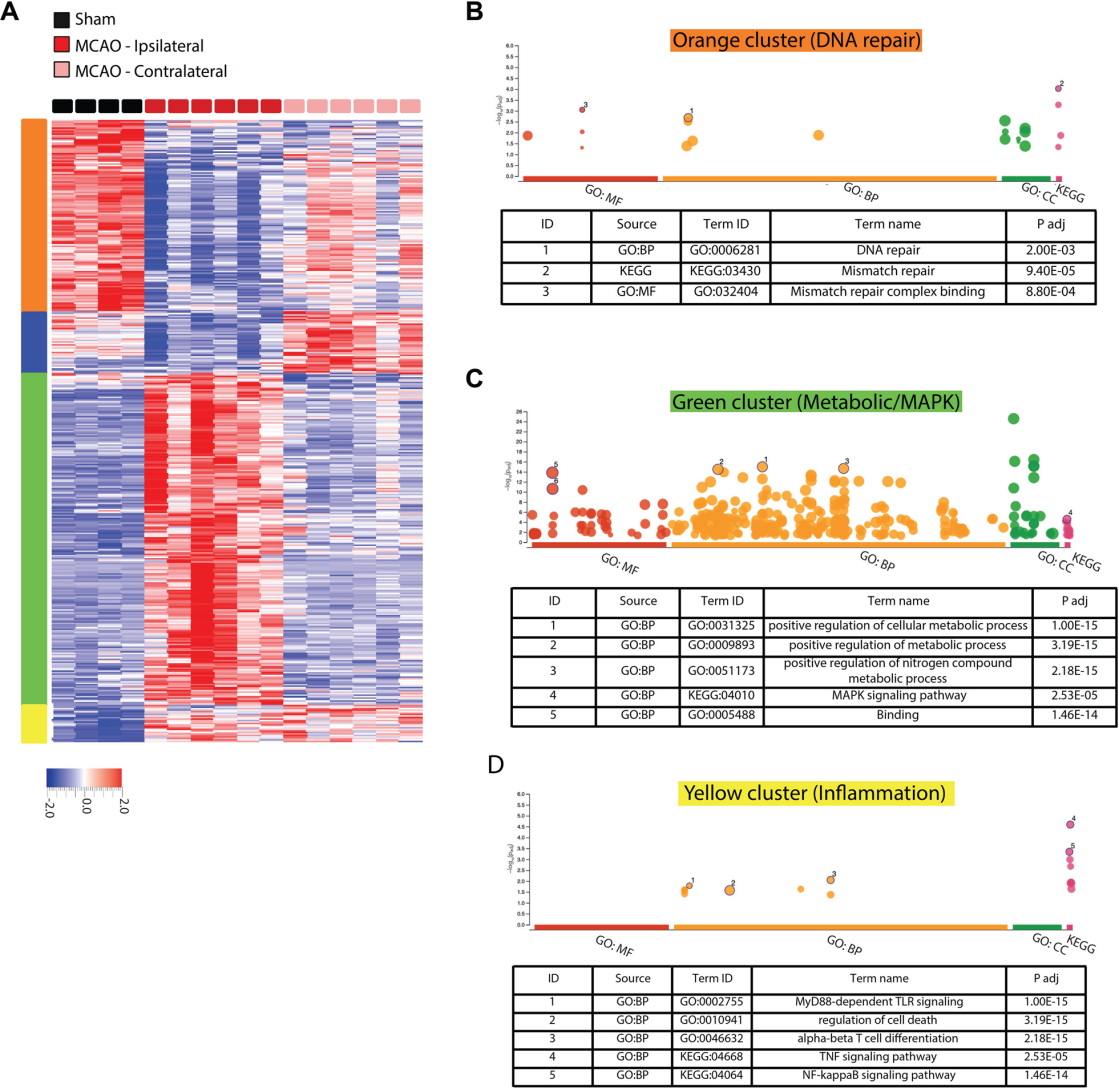
tMCAO followed by 3 h reperfusion induces robust changes in transcriptome in the CP of WT mice. **A** Gene clustering and heat map of top 1000 differentially regulated genes (*q* < 0.05) in the CP ipsilateral to tMCAO compared to the CP contralateral to tMCAO. **B**–**D** gProfiler gene ontology graphs and top GO terms related to the indicated clusters. The circle size correlates with the number of genes related to the GO term. No GO term was found related to the blue cluster. *BP* biological process, *MF* molecular function, *CC* cellular component

To understand the mechanism behind lower leukocyte infiltration in the CP of CD36 KO mice after tMCAO, RNA sequencing was performed on CPs ipsilateral and contralateral to tMCAO and transcriptome analysed at 3 h post-reperfusion time when we observed increased leukocyte accumulation in the ipsilateral CP. In WT mice, gene clustering and ontology analysis revealed that a cluster of genes related to DNA repair was significantly downregulated in ipsilateral CP compared to both the sham and contralateral CP following tMCAO (Orange cluster, Fig. 3A, B), while a cluster of genes related to metabolic pathways was upregulated (Green cluster, Fig. 3A, C). Genes in the blue cluster were downregulated in ipsilateral CP compared to both the sham and contralateral CP following tMCAO, but did not contribute to any Gene Ontology term and thus not further analysed (Fig. 3A). Interestingly, while many genes were unaffected in the contralateral CP compared to CPs in sham pups, a cluster of inflammatory genes was upregulated in both ipsilateral and contralateral CP compared to the sham group (Yellow cluster, Fig. 3A, D). This could suggest that extracellular inflammatory stimuli are circulated in the CSF affecting both ipsilateral and contralateral CP, while metabolic responses are mostly regulated by intracellular pathways on the side of tMCAO.

### CD36 deficiency attenuates changes in gene expression in the CP ipsilateral to tMCAO

**Fig. 4:**
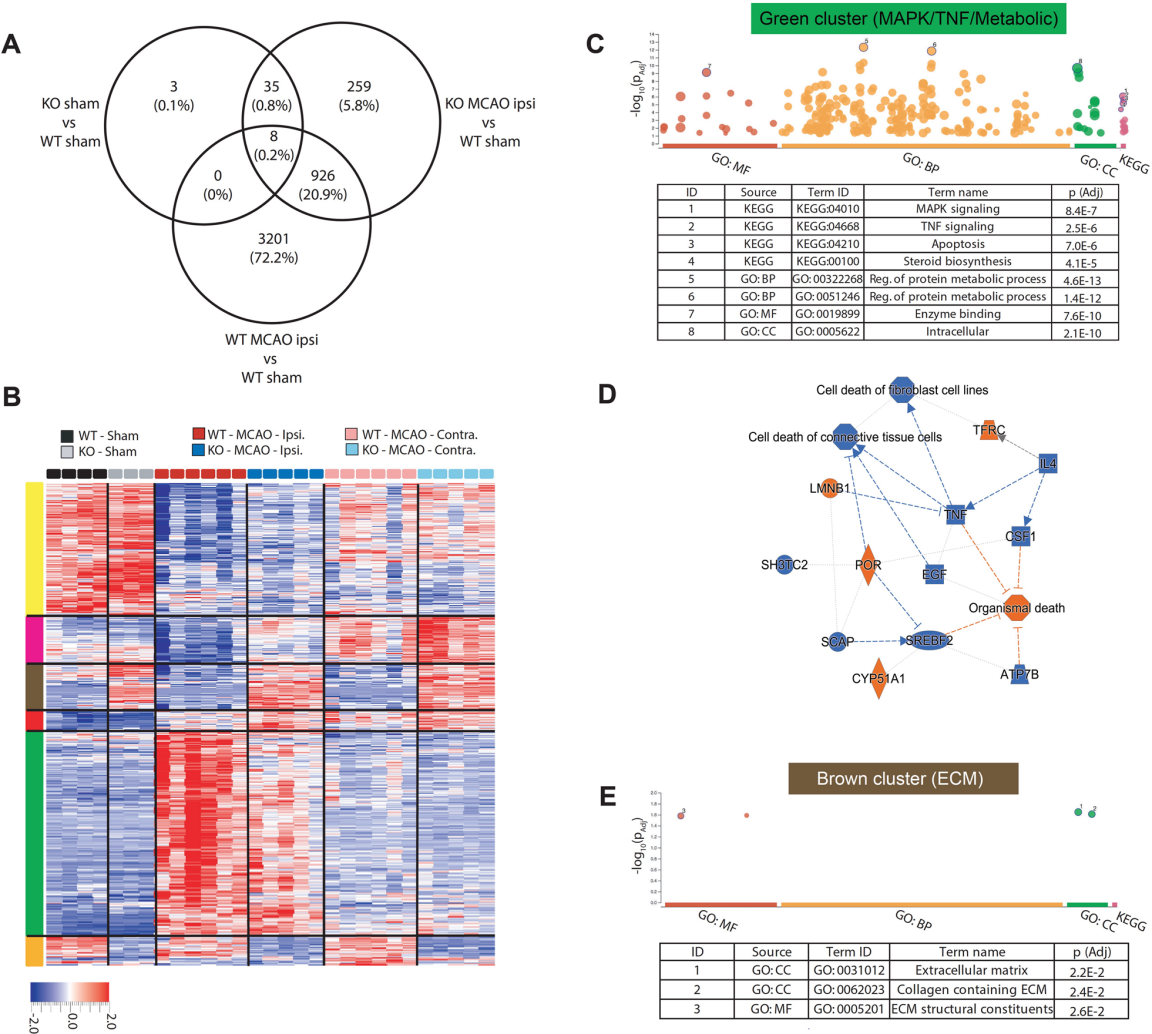
Lack of CD36 alters gene expression response in the CP of neonatal mice 3 h after tMCAO. **A** Venny diagram showing the number of differentially expressed genes (*q* < 0.05) and the number/percentage of genes shared between different analyses. **B** Gene clustering and heat map of top 1000 genes significantly (*q* < 0.05) different between groups. **C** gProfiler gene ontology graphs and top GO terms related to the green cluster. **D** Ingenuity Pathway Analysis (IPA) showing a network of pathways and key regulators related to genes in the green cluster and top canonical pathways. Only genes that are significantly different between WT and CD36 KO ipsilateral CP in the green cluster were included in IPA analysis. **E** gProfiler gene ontology graphs and top GO terms related to the brown cluster. *KO* CD36 KO, *WT* wild type, *Ipsi* ipsilateral, *Contra* contralateral, *CC* cellular component, *BP* biological process, *MF* molecular function

In sham-operated mice, expression of only 46 genes in the CP was significantly different between WT and CD36 KO mice (Fig. 4A, Additional file 1), suggesting that the baseline gene expression is comparable between the two genotypes. tMCAO followed by 3 h of reperfusion significantly altered expression of 4135 genes in ipsilateral CP compared to CP of sham-operated WT mice, while expression of only 1228 genes was altered in ipsilateral CP of CD36 KO mice (Fig. 4A). This demonstrates that while under physiological conditions transcriptome makeup in the CPs is similar in both groups, transcriptome response to tMCAO is considerably more robust in in CP in WT compared to CD36 deficient mice.

Cluster analysis was performed on top 1000 genes differentially regulated between all groups (*q* < 0.05, Fig. 4A). Three subgroups of genes demonstrated similar patterns of altered gene expression in ipsilateral and contralateral CP of WT and CD36 KO mice in response to tMCAO (gene clusters grouped by Yellow, Pink and Red vertical bars). In contrast, three clusters of genes were identified in CP ipsilateral to tMCAO that had a distinctly different gene expression pattern in CD36 KO mice compared to WT (Green, Brown and Orange, Fig. 4B). The Orange cluster gene ontology only provided a KEGG pathway related to lysosome with border significance (*p* = 0.043). The gene ontology for Green (Fig. 4C) and Brown (Fig. 4E) clusters of genes produced more robust outcomes, and therefore, we focused on these clusters as potentially more biologically significant. The Green cluster contained genes related to metabolic processes, sterol biosynthesis and cell fate signalling of MAPK and TNFa (Fig. 4C). IPA analysis of genes in this cluster predicted inhibition of TNF signalling and cell death pathways in CD36 KO ipsilateral CPs (Fig. 4D). A second cluster of genes related to the extracellular matrix (ECM, Fig. 4E) also showed markedly different pattern of expression in CD36 KO CPs compared to the WT in both ipsilateral and contralateral CP (identified by the Brown vertical bar, Fig. 4B). Overall, these data indicate that deficiency in CD36 mutes tMCAO-induced metabolic and cell fate signalling and markedly alters an ECM-related transcriptional program in the CP.

### Genetic deletion of CD36 alters mRNA and protein expression of key players in inflammation, leukocyte trafficking and ECM pathways in the CP after neonatal stroke

**Fig. 5:**
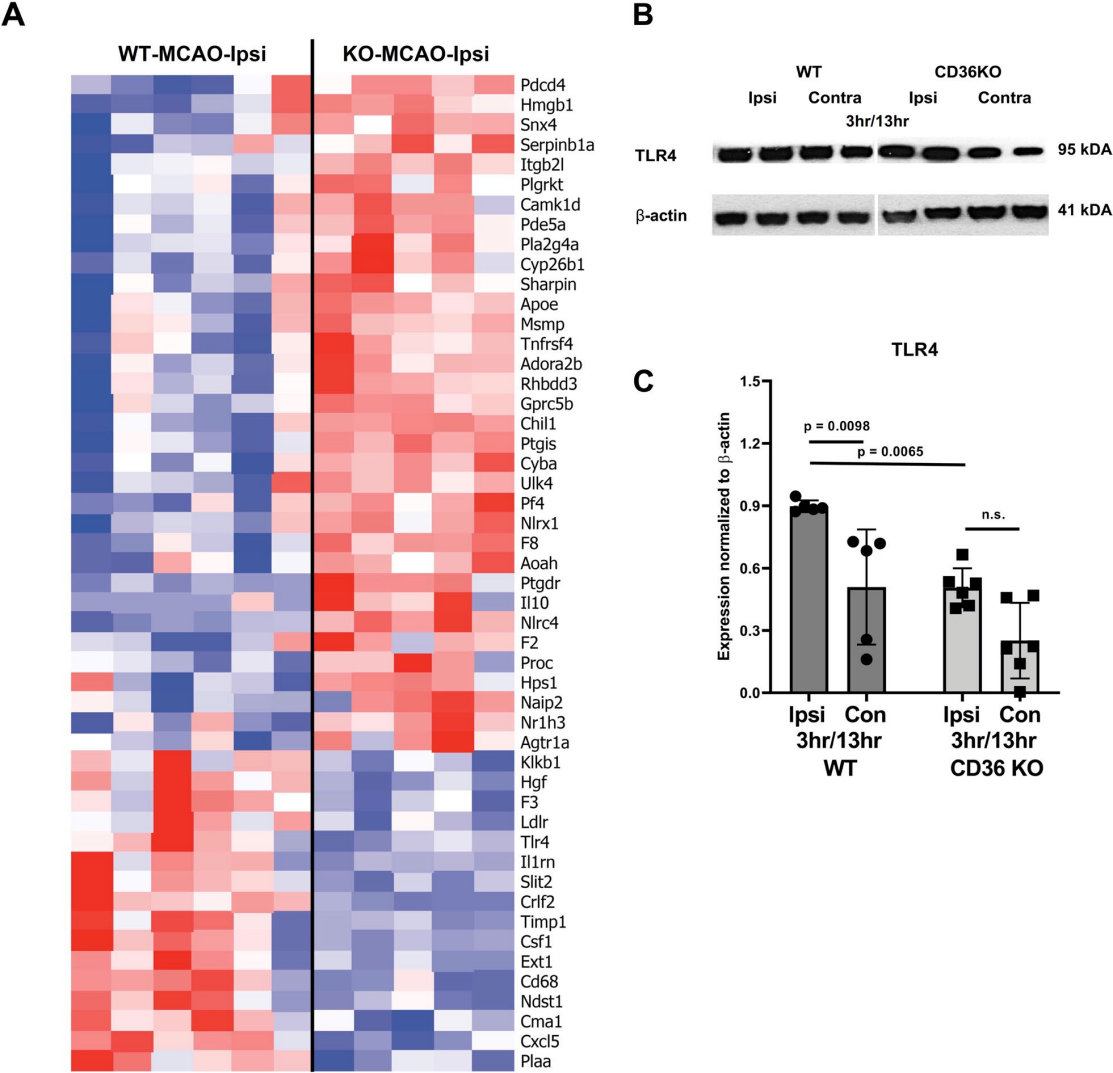
Lack of CD36 alters the inflammatory response of the CP to neonatal stroke. **A** Top 50 inflammatory response genes (GO: 0006954) significantly different in expression between WT and CD36 KO mice in the ipsilateral (ipsi) CP 3 h after reperfusion. **B—C** Western blot validation of TLR4 expression (**B**) and its quantification (**C**) in the CP of WT and CD36 KO pups 13 h after reperfusion

We then focused on changes in inflammation-related genes (obtained from Gene Ontology database, GO: 0006954) in the CP ipsilateral to tMCAO (Fig. 5A). We observed marked differences between WT and CD36 KO of multiple well-described inflammatory players in neonatal stroke and hypoxia–ischemia, including down-regulation of Tlr4, Timp1, Csf-1 and CD68. At the same time, several genes related to inflammation were up-regulated in CP of CD36 KO compared to WT mice (Fig. 5A). Among these were anti-inflammatory cytokine IL-10 and damage-associated molecular pattern, Hmgb1, as well as coagulation-related molecules, such as Pf4 and F8 (Coagulation Factor VIII). This suggests that while WT CPs presents a classic pro-inflammatory response, the CP of CD36 KO pups show a complex pattern of immune response mediated by various pro- and anti-inflammatory genes, potentially also linked to the coagulation cascade.

**Fig. 6:**
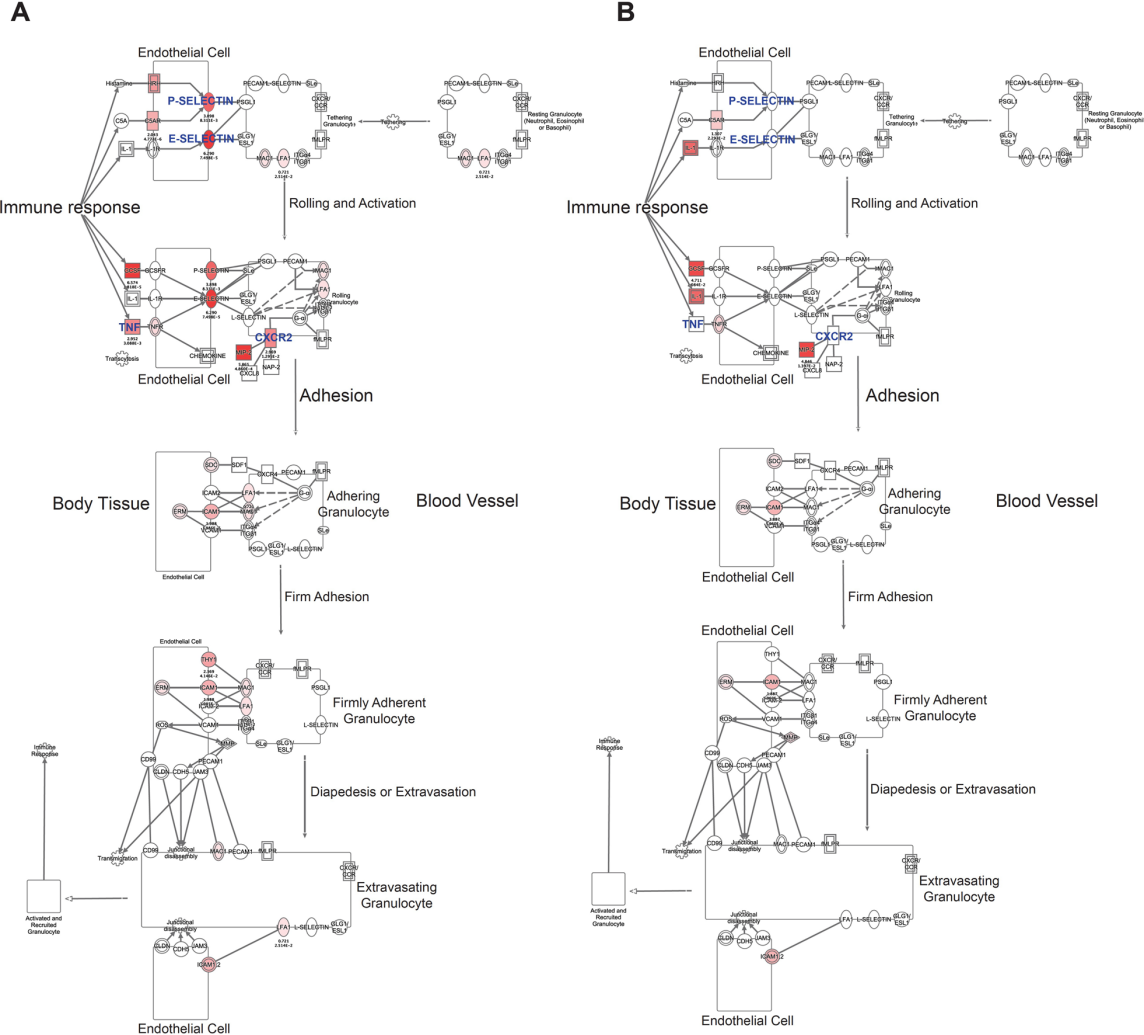
Lack of CD36 reduces expression of key genes in Granulocyte Adhesion and Diapedesis pathway in pups subjected to tMCAO followed by 3 h reperfusion. IPA showing the molecular pathway of Granulocyte Adhesion and Diapedesis overlayed with differential expression data of WT (**A**) and CD36 KO (**B**) in the CP ipsilateral to MCAO, showing lower expression of IL-1, P-selectin and E-selectin, LFA1 and MAC1 in CD36 KO CP. The red indicates gene’s upregulation and green represents downregulation

Knowing that TLR4 is one of common CD36 co-receptor partners during inflammation and cerebral ischemia, including neonatal stroke [29], we measured protein levels of TLR4 in the ipsilateral and contralateral CPs of WT and CD36 KO mice. There was a significant downregulation of TLR4 in the CP of CD36 KO pups compared to WT pups at 13 h reperfusion, corresponding to what we observed 3 h after reperfusion at the RNA level (Fig. 5B, C). To further relate our RNA data with leukocyte trafficking, we overlaid differential expression data from WT and CD36 KO mice on a network of genes involved in Granulocyte Adhesion and Diapedesis obtained from IPA. In particular, the genes involved in the rolling and activation stage were differentially expressed in WT and CD36 KO CP, with fewer changes observed in adhesion, diapedesis and extravasation steps (Fig. 6A, B). The networks showed upregulation of several key genes in these pathways, including endothelial adhesion molecules E-selectin and P-selectin in ipsilateral CP of WT (Fig. 6A), but not in CD36 KO mice (Fig. 6B). Furthermore, this network analysis showed down-regulation of TNFa, as also predicted by IPA analysis of the green cluster (Fig. 4D). Altogether, the results reveal key mediators of inflammatory response and leukocyte trafficking in the WT mice ipsilateral CP that were downregulated in the CP of CD36 KO mice.

**Fig. 7:**
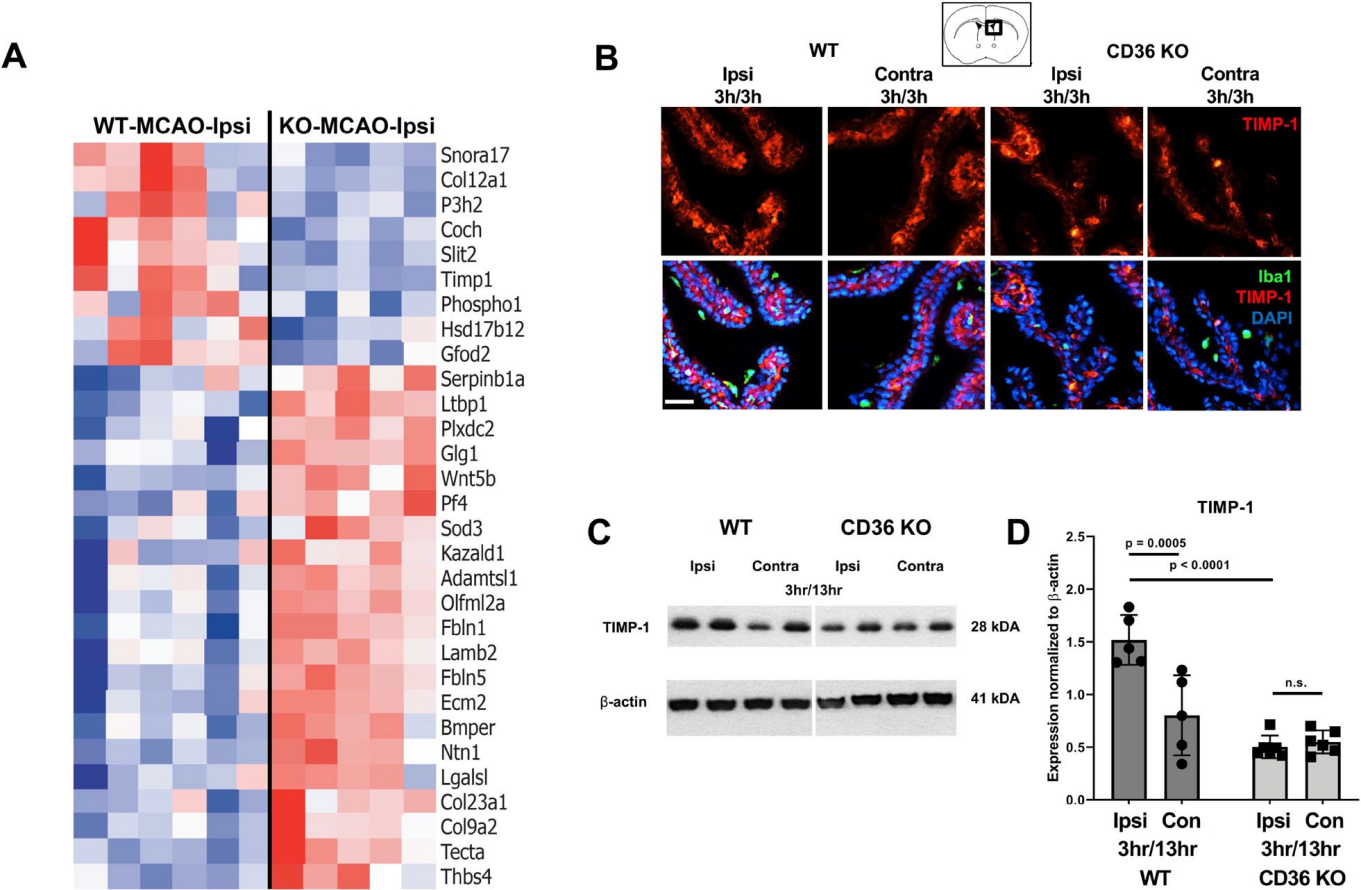
Expression of genes related to basement membrane signalling is altered in CD36 KO pups 3 h after reperfusion. **A** Top 30 basement membrane genes (GO: 0005604) with significantly different expression between WT and CD36 KO ipsilateral CP 3 h following tMCAO. **B** Representative images of CPs immunostained with DAPI (blue), Iba1 (green), and Timp1 (red) ipsilateral and contralateral to tMCAO in WT and CD36 KO mice at 3 h reperfusion. Scale bar = 50 µm. **C** and **D** Representative western blots for Timp1 and β-actin in CPs ipsilateral and contralateral to tMCAO (**C**) and its quantification (**D**) 3 h and 13 h after tMCAO. Quantification of Timp1 expression was normalized to β-actin. Two-way ANOVA with post-hoc Tukey’s Multiple Comparison test (**D**). Dots represent individual mice. Data are shown as mean ± SD. Individual *p* values are listed within figures for data that are significant

The second cluster with different pattern of gene regulation was related to ECM composition and function (Brown cluster Fig. 4A, E). Therefore, we extracted a list of genes related to the main component of ECM, basement membrane (GO: 0005604), and compared the patterns between WT and CD36 KO. Several genes were differentially expressed between ipsilateral CP of WT and CD36 KO mice (Fig. 7A). Moreover, differential expression data showed that several members of matrix metalloprotease proteins (MMPs; e.g., Mmp3, Mmp17, Mmp8) as well as metalloendopeptidase family of proteins such as ADAMs (e.g., Adamtsl2, Adamts9, Adamts8, Adamts11) were only upregulated in WT ipsilateral CP and not in the CD36 KO CP (Additional file 1). Interestingly, expression of the negative regulator of MMPs and ADAMs, Timp1, was significantly higher in ipsilateral CP of WT than ipsilateral CP of CD36 KO mice, suggesting a negative feedback mechanism (Fig. 7A).

Since our gene analyses revealed Timp1 as an ECM and inflammation associated gene modified by the lack of CD36 after tMCAO, we next evaluated whether CD36 influenced protein expression of Timp1 after tMCAO. Immunostaining showed Timp1 expression in the ipsilateral and contralateral CPs at the level of the lateral ventricles of both groups (Fig. 7B). In the CP, Timp1 did not co-localize with Iba1^+^ and was mainly observed in the CP epithelium. There was minimal Timp1 staining in the brain parenchyma and in vascular cells (data not shown).

As immunofluorescence staining of Timp1 expressing CP epithelium appeared more preserved in WT CPs compared to CD36 KO CPs, we performed Western blot on isolated CPs to quantify Timp1 protein expression at 13 h reperfusion following tMCAO. Compared to the contralateral CP, Timp1 protein expression was significantly higher in the ipsilateral CP in WT mice, but was unchanged in CD36 KO mice (Fig. 7C, D). Consistently, and confirming our RNA data, Timp1 expression in the ipsilateral CP of WT mice was significantly higher compared to CD36 KO mice. Overall, these results show that lack of CD36 restricts tMCAO-induced expression of Timp1 in the CP epithelium, pointing to modulatory effects of CD36 on ECM signalling in the CP.

### CD36 deficiency alters the dynamics of myeloid signalling in the CP from an inflammatory to an anti-inflammatory phenotype

**Fig. 8:**
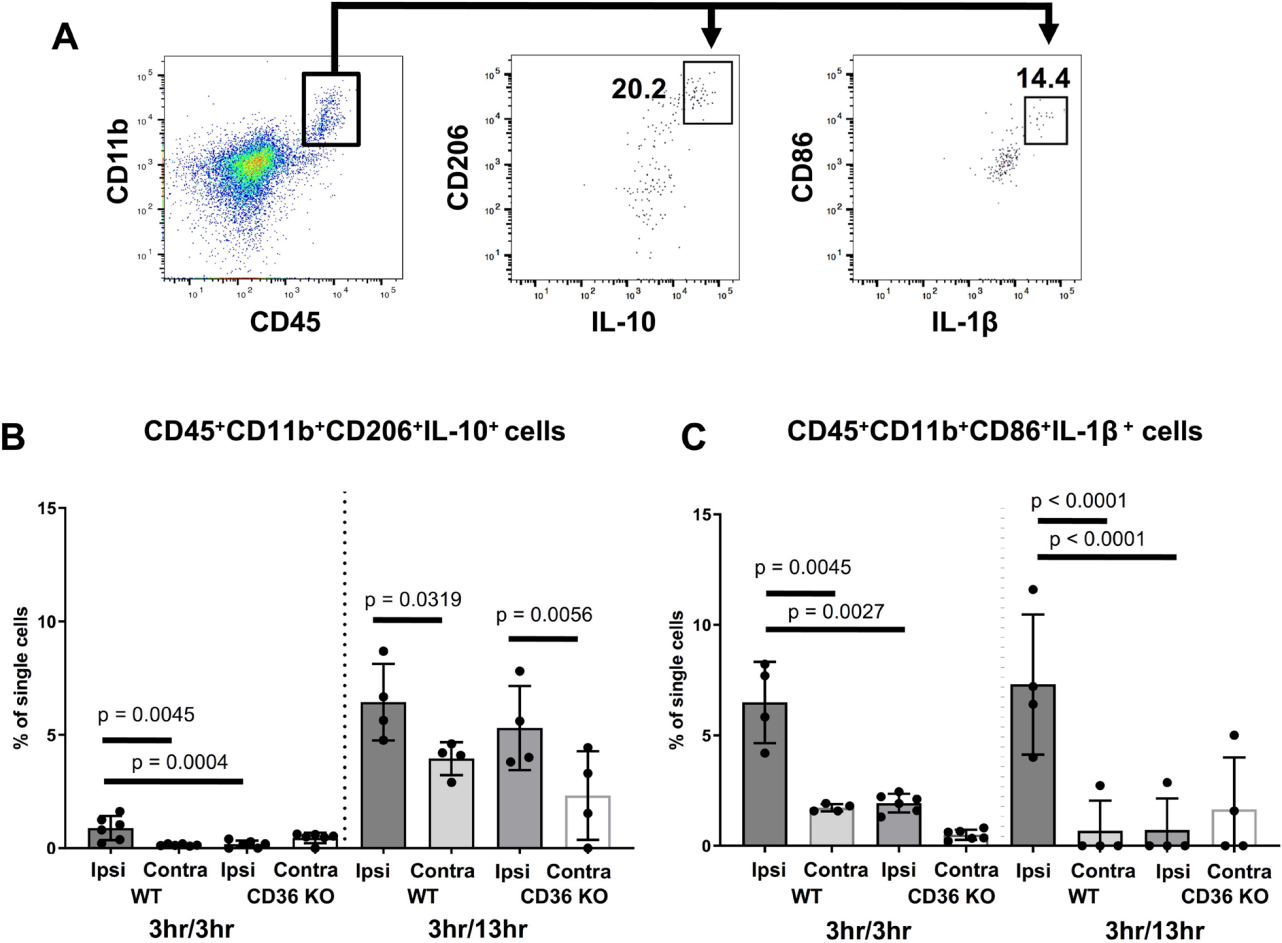
CD36 deficient mice exhibit altered dynamics of anti- and pro-inflammatory myeloid phenotypes in the CP following tMCAO. **A** Gating strategy for CD11b^+^CD45^+^CD206^+^IL-10^+^ and CD11b^+^CD45^+^CD86^+^IL-1β^+^cells. Quantification of CD11b^+^CD45^+^CD206^+^IL-10 + cells (**B**) and CD11b^+^CD45^+^CD86^+^IL-1β^+^cells (**C**) ipsilateral (ipsi) and contralateral (contra) to tMCAO in WT and CD36 KO mice at 3 h and 13 h reperfusion. Two-way ANOVA with post-hoc Tukey’s Multiple Comparison test was performed to compare groups with multiple independent variables. Dots represent individual mice. Data are shown as mean ± SD. Individual *p* values are listed within figures for data that are significant

Based on our findings that lack of CD36 influences inflammatory gene signatures as well as TLR4 and Timp1 protein expression in the CP early after reperfusion, we evaluated how CD36 impacts the cytokine expression in infiltrating myeloid cells. To that end, we compared anti- and pro- inflammatory myeloid cell phenotypes in the CP by measuring the number of CD45^+^CD11b^+^CD206^+^IL-10^+^ and CD45^+^CD11b^+^CD86^+^IL-1β^+^ cells (Fig. 8A demonstrates gating strategy). tMCAO triggered a small but significant increase in the number of CD45^+^CD11b^+^CD206^+^IL-10^+^ cells in the ipsilateral CP of WT mice compared to respective contralateral CP and ipsilateral CP of CD36 KO mice at 3 h reperfusion. By 13 h reperfusion, the number of CD45^+^CD11b^+^CD206^+^IL-10^+^ cells was increased in ipsilateral CP of both WT and CD36 KO mice compared to their contralateral counterparts (Fig. 8B), demonstrating the evolving accumulation of cells with anti-inflammatory phenotypes within hours after reperfusion.

At the same time, tMCAO triggered significant accumulation of CD45^+^CD11b^+^CD86^+^IL-1β^+^ cells in the ipsilateral CP of WT mice compared to that in contralateral CP at 3 h reperfusion, with this increase maintained at 13 h reperfusion. By contrast, CD36 KO mice demonstrated halted numbers of CD45^+^CD11b^+^CD86^+^IL-1β^+^ cells at both post-reperfusion timepoints compared to WT mice (Fig. 8C). These data demonstrate that compared to WT, CD36 KO mice display attenuated number of toxic cell phenotypes resulting in an anti-inflammatory skewed response at 13 h reperfusion.

## Discussion

We demonstrate for the first time that CD36 mediates step-wise leukocyte recruitment to the CP early after tMCAO in neonatal mice, first with a predominant increase in inflammatory monocytes and neutrophils, followed by a delayed increase in the accumulation of patrolling monocytes. These effects parallel early increase in the number of CD45^hi^CD11b^+^ cells in ischemic-reperfused brain parenchyma. RNA sequencing analysis in the CPs reveals that lack of CD36 attenuates the inflammatory gene profile induced by ischemia–reperfusion and alters the makeup of the CP ECM. Together, these data suggest cooperation between peripheral and brain CD36—mediated signalling following neonatal stroke via the CP.

CD36 was shown to contribute to stroke, neurodegenerative diseases and dementia and is being considered as a therapeutic target during adulthood and aging [13, 17, 30–34]. CD36 deficiency led to protection of adult mice from injury after tMCAO via several mechanisms that include reduced superoxide accumulation in activated microglia/macrophages and in the vasculature [13], a shift toward anti-oxidative [30] and anti-inflammatory [32] brain status and attenuation in inflammasome activation [34]. In the adult, synergy of CD36 signalling between the periphery and the injured brain after stroke [35], along with induced BBB leakage [36], has been demonstrated. Conditional deletion of CD36 either in microglia or endothelium reduced ischemic injury in adult mice, demonstrating the injurious involvement of CD36 in both cell types [34]. While these findings attest to an injurious role of CD36, its role in monocytes and macrophages in mediating phagocytosis during the resolution phase of stroke has also been demonstrated [37], suggesting potentially beneficial aspects of CD36 signalling. Interestingly, only preventative, but not post-stroke inhibition of CD36 attenuated brain swelling in hyperlipidemic stroke [38].

In a neonatal tMCAO model, we previously showed that while the magnitude of injury on diffusion-weighted MRI during MCAO was similar in WT and CD36 KO, lack of CD36 substantially increased incidence of severe acute injury, in part by omitting CD36-dependent phagocytosis of dead neurons 24 h after reperfusion [21]. More severe injury in CD36 KO pups was associated with increased gene and protein levels of monocyte/microglial chemoattractant MCP-1 and increased number of CD11b^+^TLR2^+^ cells in injured regions but, interestingly, several aspects of CD36 inflammatory signalling reported in adult stroke were not present. For example, NfkB activation and superoxide accumulation in injured regions of neonates were unaffected by lack of CD36 [21], suggesting brain maturation-dependent inflammatory responses of CD36.

Here, in WT mice, we report rapid accumulation of multiple myeloid subtypes at 3 and 13 h after reperfusion in the CP ipsilateral to tMCAO as well as parallel early increase in the number of CD45^+^/CD11b^+^ cells in ischemic-reperfused regions. In the CP of CD36 KO pups, in turn, accumulation of CD45^hi^CD11b^+^ cells is significantly attenuated, including CD45^hi^CD11b^+^Ly6c^hi^ inflammatory monocytes and CD45^+^11b^+^Ly6c^int^Ly6g^+^ neutrophils. Reduction of leukocyte trafficking might be due to inhibition of certain inflammatory and chemotactic pathways required for leukocyte recruitment to the inflammation site, as suggested by IPA data. Examination of the phenotypes of cells accumulated in the ipsilateral CP, inflammatory vs. anti-inflammatory, also showed largely unaffected numbers of CD45^+^CD11b^+^CD206^+^IL-10^+^ cells but lower numbers of inflammatory CD45^+^CD11b^+^CD86^+^IL-1β^+^ cells in CD36 KO pups compared to WT pups at both post-reperfusion timepoints, suggesting that lack of CD36 is anti-inflammatory in the CP early after injury. In the ischemic-reperfused parenchyma, attenuation of CD45^hi^CD11b^+^ cells in CD36 KO pups is transient at 3 h, as the overall numbers of myeloid cells in the ipsilateral cortex at 13 h do not depend on CD36 presence. Nonetheless, the accumulation of both inflammatory monocytes and neutrophils are lower at both timepoints in the injured cortex of WT mice compared to CD36 KO mice.

Considering that the dynamic interactions at the CSF-brain interface early after reperfusion can contribute to the forming/evolving inflammatory response, we examined gene expression in the CP at this early timepoint after tMCAO, at 3 h reperfusion. Although the CP is not directly affected by cerebral blood flow reduction in this model, tMCAO itself induced rapid profound changes in gene expression in the CP ipsilateral to the occlusion. Most notably, we demonstrate downregulation of genes related to DNA repair and activation of inflammation and metabolic processes. Furthermore, while under more basal conditions (sham), gene expression is similar in both WT and CD36 KO CPs, the magnitude of significantly regulated genes in ipsilateral CP is muted in CD36 KO, i.e., ~ threefold lower than in WT pups (1228 vs. 4135 genes), consistent with attenuated leukocyte accumulation in CP of CD36 KO mice after tMCAO. The inflammatory and metabolic pathways are among those that were most prominently affected. Gene clustering analysis and IPA suggested that genetic deletion of CD36 diminishes several key inflammatory pathways including expression of one of the key upstream inflammatory receptors, TLR4, which signals in part by formation of TLR4-TLR6 complex in response to sterile inflammation [17, 18]. Consistent with lower gene expression, protein expression of TLR4 was significantly lower in CP in ischemic-reperfused CD36 KO than in WT, not only validating RNAseq data but also demonstrating that lack of its co-receptor, CD36, attenuates TLR4 upregulation and TLR4–mediated effects. These data expand the importance of CD36-TLR2/4/6 interactions for inflammatory signalling from adult [17] to neonatal brain. Increased expression of anti-inflammatory genes and corresponding proteins, such as a classic anti-inflammatory cytokine IL-10, may serve as another modulator of leukocyte trafficking, especially when associated with reduced IL-1β levels in cells of monocyte lineage.

Recruitment of leukocytes to the inflammation site requires rearrangements in the cytoskeleton of leukocytes and the ECM of endothelium and epithelium of affected tissue [40]. We previously showed that TLR2-mediated leukocyte trafficking through the CP leads to structural changes in basement membrane of CP epithelium and transcriptomic changes in cytoskeleton genes [27, 41]. Here, we show that lack of CD36 markedly alters expression of several genes related to the ECM following tMCAO, including an MMP inhibitor Timp1, as well as key endothelial adhesion molecules. While ECM can act as a barrier to invading cells, it can catalyse leukocyte adhesion via interaction of adhesion molecules expressed on leukocytes (e.g., integrins) as well as chemokines [42, 43]. Several enzymes with peptidase activity (MMPs and ADAMs) in the ECM were upregulated after tMCAO in WT CP, but not in the CD36 KO, which coincides with upregulation of the MMP inhibitor, Timp1, in the CP of WT but not CD36 KO mice, suggesting a negative feedback loop.

A number of findings warrant further investigation. For example, CD36 itself can act as a pattern recognition receptor that responds to ligands such as OxLDL and activates inflammasome pathways [34, 44]. It is possible that oxLDL and other endogenous ligands are released after stroke, activating CD36-inflammasome signalling in the CP and contributing to inflammatory response [19]. In support, expression of Nlrp3, a key inflammasome pathway component, was reduced in CD36 KO mice. Our data show very dynamic changes in the patterns of myeloid cell accumulation between 3 and 13 h after reperfusion, which may depend on the magnitude of building inflammatory response in the CSF and in the parenchyma, therefore, relationships between early events in the CP and more severe injury in the parenchyma of CD36 KO are not trivial. In addition, intriguingly, expression of some genes within the inflammatory response GO term with relation to coagulation were higher in CP of CD36 KO mice. Recently, platelets’ CD36 has been shown to promote thrombosis [39]. Therefore, upregulation of some coagulation genes in CD36 KO may be a compensatory mechanism [45]. Another unexplored aspect in our study is cell-type specific effect of CD36 signalling in multiple cell types. In adult brain injury models, microglial CD36 has been proposed as a key determinant of post-ischemic IL-1β production in mice by regulating caspase-1 activity, whereas endothelial CD36 is supposedly needed for the full expression of the endothelial activation induced by IL-1β [34]. While this study does not allow distinguishing effects in individual cell types due to global CD36 deletion, future studies entail understanding how the presence of CD36 on specific cell types influences neonatal neuroinflammation. Translational impact of our study is demonstration of brain maturation-dependent effects of CD36 after stroke. Further implications of CD36 in neonatal stroke as compared to adult stroke are to be revealed by the use of pharmacological inhibitors/activators of CD36 in the neonatal stroke model.

## Conclusion

Neonatal tMCAO induces a rapid inflammatory gene response, particularly to the ipsilateral CP, a region not directly affected by stroke in this model. Comparisons of global gene expression in the CPs between WT and CD36 KO neonates revealed the much broader and marked changes in WT compared to CD36 KO after tMCAO, including significantly smaller magnitude of inflammatory gene responses in the knockouts. Our findings illustrate that CD36 modulates immune cell recruitment through the CP by regulation of inflammatory, metabolic, and ECM-related mechanisms as CD36 deficient mice display muted transcriptional changes in the ipsilateral CP and decreased peripheral immune cell accumulation. Overall, our study highlights the importance of CD36 and the CP in regulating neuroinflammatory responses in the developing brain.

## Supplementary Information

**Additional file 1.** The list of RNA sequencing, normalized count and differential expression data for naive/sham pups and pups subjected to tMCAO.

## Data Availability

All generated or analysed data in this study are included in the manuscript.

## Abbreviations

BBB: Blood–brain barrier
CP: Choroid plexus
ECM: Extracellular matrix
FOV: Field of view
KO: Knockout
P9: Postnatal day 9
oxLDL: Low-density lipoprotein
tMCAO: Transient middle cerebral artery occlusion
TLR: Toll-like receptors
WT: Wild type
